# Evaluation of CSTB and DMBT1 expression in saliva of gastric cancer patients and controls

**DOI:** 10.1186/s12885-022-09570-9

**Published:** 2022-04-30

**Authors:** Maryam Koopaie, Marjan Ghafourian, Soheila Manifar, Shima Younespour, Mansour Davoudi, Sajad Kolahdooz, Mohammad Shirkhoda

**Affiliations:** 1 Department of Oral Medicine, School of Dentistry, Tehran University of Medical Sciences, Tehran, Iran; 2 Department of Oral Medicine, School of Dentistry, Tehran University of Medical Sciences, Tehran, Iran; 3 Department of Oral Medicine, Imam Khomeini Hospital, Tehran University of Medical Sciences, North Kargar St, P.O.Box:14395-433, Tehran, 14399-55991 Iran; 4 Dentistry Research Institute, Tehran University of Medical Sciences, Tehran, Iran; 5 Department of Computer Science and Engineering and IT, School of Electrical and Computer Engineering, Shiraz University, Shiraz, Iran; 6 Universal Scientific Education and Research Network (USERN), Tehran, Iran; 7 Department of General Oncology, Cancer Research Center, Cancer Institute of Iran, Tehran University of Medical Sciences, Tehran, Iran

**Keywords:** CSTB, DMBT1, Saliva, Gastric cancer, Machine learning

## Abstract

**Background:**

Gastric cancer (GC) is the fifth most common cancer and the third cause of cancer deaths globally, with late diagnosis, low survival rate, and poor prognosis. This case-control study aimed to evaluate the expression of cystatin B (CSTB) and deleted in malignant brain tumor 1 (DMBT1) in the saliva of GC patients with healthy individuals to construct diagnostic algorithms using statistical analysis and machine learning methods.

**Methods:**

Demographic data, clinical characteristics, and food intake habits of the case and control group were gathered through a standard checklist. Unstimulated whole saliva samples were taken from 31 healthy individuals and 31 GC patients. Through ELISA test and statistical analysis, the expression of salivary CSTB and DMBT1 proteins was evaluated. To construct diagnostic algorithms, we used the machine learning method.

**Results:**

The mean salivary expression of CSTB in GC patients was significantly lower (115.55 ± 7.06, *p* = 0.001), and the mean salivary expression of DMBT1 in GC patients was significantly higher (171.88 ± 39.67, *p* = 0.002) than the control.

Multiple linear regression analysis demonstrated that GC was significantly correlated with high levels of DMBT1 after controlling the effects of age of participants (R^2^ = 0.20, *p* < 0.001).

Considering salivary CSTB greater than 119.06 ng/mL as an optimal cut-off value, the sensitivity and specificity of CSTB in the diagnosis of GC were 83.87 and 70.97%, respectively. The area under the ROC curve was calculated as 0.728. The optimal cut-off value of DMBT1 for differentiating GC patients from controls was greater than 146.33 ng/mL (sensitivity = 80.65% and specificity = 64.52%). The area under the ROC curve was up to 0.741.

As a result of the machine learning method, the area under the receiver-operating characteristic curve for the diagnostic ability of CSTB, DMBT1, demographic data, clinical characteristics, and food intake habits was 0.95. The machine learning model’s sensitivity, specificity, and accuracy were 100, 70.8, and 80.5%, respectively.

**Conclusion:**

Salivary levels of DMBT1 and CSTB may be accurate in diagnosing GCs. Machine learning analyses using salivary biomarkers, demographic, clinical, and nutrition habits data simultaneously could provide affordability models with acceptable accuracy for differentiation of GC by a cost-effective and non-invasive method.

## Introduction

Gastric cancer (GC) is the fifth most common malignancy in the world. It is the third most common cause of cancer deaths [1]. This cancer is 2 - 3fold more prevalent in men than women and the death rate is more incident in men [2]. The incidence of GC varies according to the geographical region and culture of each region. So that more than 50% of new cases occur in developing countries, including Iran [3]. The most common type of cancer incidence and cancer mortality in men of Iranian people was GC [2, 3]. GC is a multifactorial disease caused by a combination of environmental factors and genetic changes [4, 5]. Environmental risk factors for this disease include smoking [6], alcohol [7], high salt intake [8], nitrite and nitrate in some foods [9], including processed meats, high consumption of red meat smoked foods, low consumption of raw fruits and vegetables containing vitamin C and antioxidants [10, 11], overweight and obesity [12, 13], *Helicobacter pylori* [14, 15] and low socioeconomic conditions [16], including low education and low income [17–19]. Approximately 90% of gastric cancers are adenocarcinomas. Non-Hodgkin lymphomas and leiomyosarcomas make up the remaining 10% [20, 21].

Photofluorography, serum pepsinogen concentration, serum ghrelin (low serum ghrelin may indicate a high risk of GC), gastrin 17, and gastric wall cell antibodies (associated with an increased risk of atrophic gastritis, which may play a role in GC) are among the non-invasive ways of screening for stomach cancer to date [22]. Endoscopic screening is cost-effective in high-prevalence areas, but in moderate-risk populations, there is no evidence that it is effective or cost-effective [23]. In addition, endoscopy is an invasive procedure in which the risk of bleeding, mucosa perforation, and death has been reported. Imaging is also used to diagnose this cancer. Including computed tomography (CT), magnetic resonance imaging (MRI^)^, and positron emission tomography (PET^)^, each of which is used for a specific purpose [24]. The limitations of these advanced imaging technologies are the lack of widespread access, training issues in the interpretation of these “advanced” images technique, selection of imaging acquisition parameters, and their diagnostic accuracies [25, 26]. Although machine learning and artificial intelligence have led to advances in diagnostic imaging techniques, there are still challenges to the early detection of GC [27–29]. Prevention of GC may be achieved through primary prevention by reducing the incidence of GC or by using secondary prevention by early detection, identifying, and treating the disease in its early stages [22, 30]. Despite significant improvements in the survival of GC patients in recent decades, GC is often diagnosed at an advanced stage and has a poor prognosis due to the high prevalence of recurrence [31–34]. Since GC is symptomatic at high levels, early detection using effective screening methods is important in reducing mortality.

Biomarkers are factors that are objectively measured and evaluated as indicators of natural biological processes, pathogenic processes, or drug responses to a therapeutic intervention [32, 35–37]. Saliva is one of the most complex biological fluids in the body, reflecting a wide range of physiological conditions in the body [38, 39]. Compared to blood sampling or biopsy, using saliva has advantages, including accessible collection and storage, less invasiveness, cost-effectiveness, and no need for specialized equipment [40]. In various studies, salivary proteins have been used as potential diagnostic markers and monitor the prognosis of disease, patient survival, and treatment [41–43].

Cystatin B (CSTB) is a protein structure encoded by the CSTB gene that acts as an intracellular thiol protease inhibitor [44]. This gene is located on chromosome 21q22.3 [45]. This protein belongs to the large family of cystatins (type two), which can form dimers stabilized by non-covalent forces and inhibit Papain and Cathepsins L, H and B, and is thought to play a role in protecting protease leakage from lysozymes [46, 47]. Deleted in malignant brain tumor 1 (DMBT1) is a tumor-inhibiting gene located on chromosome 10q25.3-q26.1 to its inactivation in several medulloblastoma cell lines in comparison with normal cells [48–51]. It plays an important role in some biological reactions, such as the innate immune system and inflammation and the recognition and accumulation of bacteria by binding to various pathogens and host molecules. This protein may act as an epithelial differentiating factor and contribute to the polarization of epithelial cells. The DMBT1 protein is encoded by the DMBT1 gene and is a scavenger receptor cysteine-rich (SRCR^)^ family [48, 52].

Nowadays, machine learning in healthcare is becoming widely used [53]. Machine learning methods help us develop computer algorithms that can consider a set of variables and their complicated relationships to accomplish specific tasks such as modeling, classification, and regression. Despite efforts to use artificial intelligence in the image-based diagnosis of GC [28, 54], artificial intelligence methods in the analysis and modeling of GC biomarkers have been limited. This study aims to evaluate the application of salivary levels of CSTB and DMBT1 in GC diagnosis, considering the importance of early diagnosis of GC through convenient and noninvasive methods. This paper purpose using statistical analysis and machine learning methods to construct a GC diagnostic algorithm based on the salivary levels of CSTB and DMBT1, demographic data, clinical characteristics, and food intake habits data.

## Methods

### Ethical statement

This study was approved by the Tehran University of Medical Sciences Ethical Committee (ethical code: IR.TUMS.DENTISTRY.REC.1398.003). After describing the study objectives, all participants signed the informed consent before participating in this study. All methods were performed in accordance with the relevant guidelines and regulations.

### Samples

**Fig. 1:**
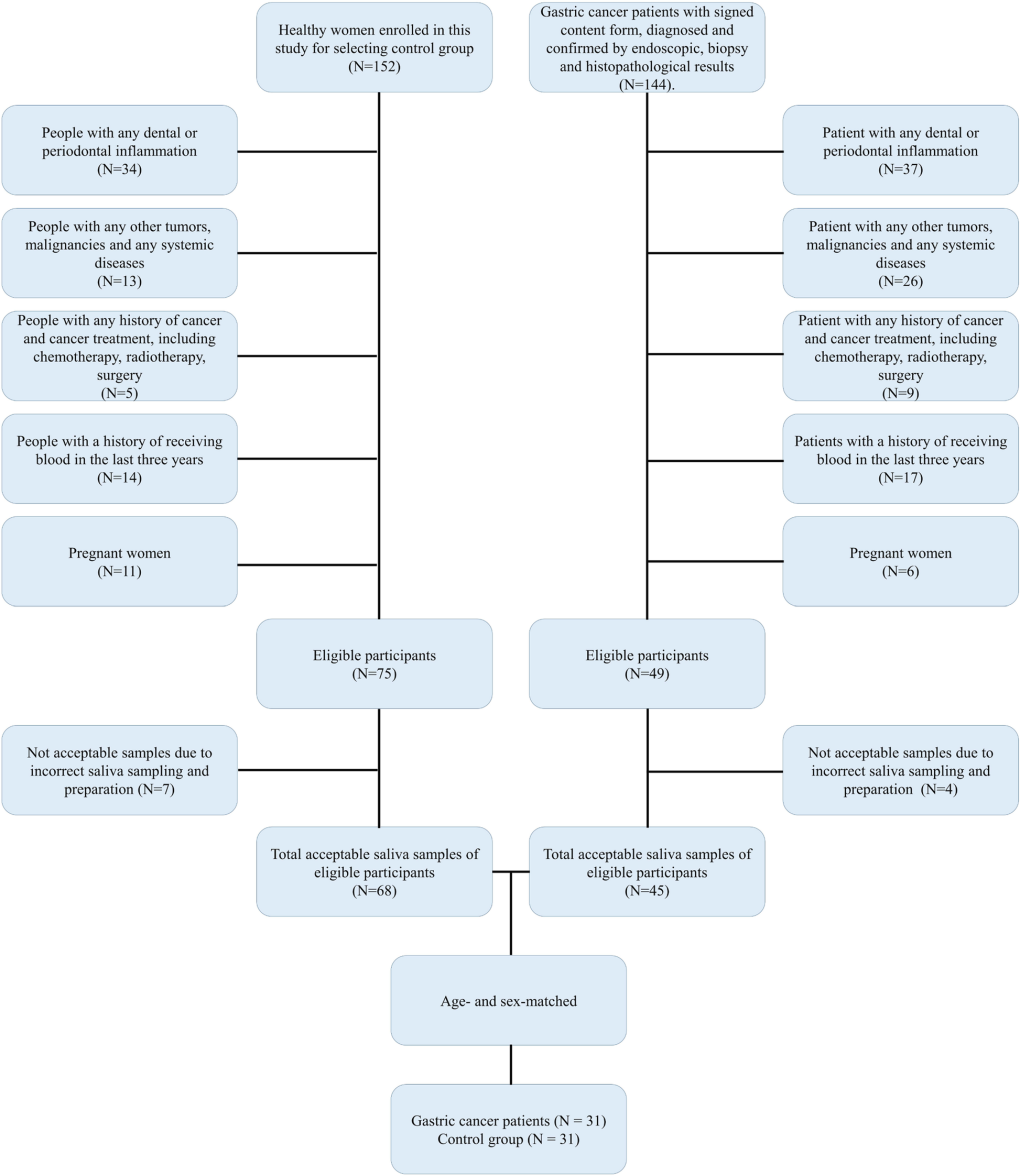
STARD flow diagram of the gastric cancer cases and control

This case-control study was undertaken on 31 healthy individuals and 31 GC (adenocarcinoma) patients in early stages, referred to Imam Khomeini Hospital in Tehran. They have been diagnosed with GC by a gastroenterologist based on histopathological and endoscopic examination. The exclusion criteria for subjects were as follows: 1) Patients with known active dental and periodontal infections. 2) Patients with a known history of any other tumors and malignancies and any obvious inflammatory diseases such as liver cirrhosis, chronic renal disease, diabetes mellitus, and also any systemic diseases. 3) Patients with a known history of any surgical operations, chemotherapy, or radiotherapy before collecting saliva. 4) Patients with a history of receiving blood in the last 3 years. 5) Pregnant women. The control group was selected from healthy individuals referred to Imam Khomeini Hospital in Tehran for routine medical checkups. The enrolled people in control groups also had no active mouth infections, inflammation, malignancies, and systemic diseases. Pregnant women, people with a history of receiving blood in the last 3 years, and people with any history of cancer treatment were excluded from the control group. Gastric cancer patients and control were age- and sex-matched (Fig. 1).

All participants were asked to carefully respond to a valid, uniform, and standardized checklist to report their demographic characteristics (gender, age, education level, and occupational stress) and habits that possibly affect GC progression. To ensure the validity and completeness of the responses, one of the trained authors supervised the completion of each questionnaire, which only explained items neutrally when necessary but did not offer any directive or indicative clues.

Participants’ occupation was classified into three groups; high-stress level, moderate stress level, and low-stress level [55]. Farmers, manual laborers, and the unemployed are categorized in high-stress level, because of low income, commercial stress, and physical stress. Sales clerks, workers in service industries, security guards, and workers in transportation or communication industries are categorized in the moderate-stress level group. Professionals, administrators, and office clerks are categorized into the low-stress level group [55]. Smoking habit is defined as current smokers who are intermittent smoker (1 > cigarettes per day (CPD)) or light smoker (1–10 CPD), moderate smoker (11–19 CPD), or heavy smoker (20 < CPD) [56]. All participants were further requested to report a positive history of gastroesophageal reflux disease (GERD), gastric ulcer, anemia, type of patient care (inpatient or outpatient), history of abdominal radiotherapy, frequency score (FS) for intake of vegetables/fruit, fast food, salty fish, preference score for salty taste, sour taste and spicy taste. FS was defined as 0, never; 1, 1 ≥ time per month; 2, 2–3 times per month; 3, 1–2 times per week; 4, 3–4 times per week; 5, 5–6 times per week; 6, 1 time per day; 7, 2 times per day; 8, 3 times per day; 9, 4 ≤ times per day. FS equal to 4 and higher were considered positive [55]. Preference score ranged from 1 (extremely dislike) to 7 (extremely like) with an increment of 1. Like and extremely like were considered positive [57].

### Saliva collection

In order to prevent the possible effects of circadian rhythm changes on salivary secretions, saliva sampling was performed from 9:00 to 11:00 am. Participants were asked to abstain from eating, drinking, smoking, and oral hygiene for 90 min before sampling to avoid salivary irritation. After dental and periodontal examination, sampling of whole non-stimulated saliva without mechanical and chemical stimulation was performed by spitting method. The person was asked to collect his saliva for 5 to 15 min at 60-s intervals and pour saliva into pre-weighed sterile containers.

### Determination of salivary CSTB and DMBT1 levels

Saliva samples were stored at − 80 °C until enzyme-linked immunosorbent assay (ELISA) examination based on the biotin double antibody sandwich technology. ELISA test was performed by 96-test ZellBio-GmbH human cystatin B (CSTB) ELISA kit (Cat. ZB-2809–H9648, ZellBio GmbH, Ulm, Germany) and 96 test ZellBio-GmbH human deleted in malignant brain tumors 1 (DMBT1) ELISA Kit (Cat. ZB-2955-H9648, ZellBio GmbH, Ulm, Germany) for measurement of the salivary level of CSTB and DMBT1, respectively, according to the manufacturer’s instructions. CSTB and DMBT1 proteins were added to the wells, precoated with anti-human CSTB and DMBT1 monoclonal antibodies. Then, anti- CSTB and DMBT1 antibodies were added and labeled with biotin to combine with streptavidin-HRP, forming an immune complex. The assay range of the ELISA kit for CSTB and DMBT1 was 50 ng/ml - 1600 ng/ml, and the sensitivity was 2.5 ng/ml. The absorbance of the samples was measured using Hyperion ELISA microplate reader. The concentrations of CSTB and DMBT1 were determined by spectrometer software based on standard curves, and all measurement procedures were repeated three times for each sample, and the mean value was reported.

### Statistical analysis methods

Statistical analysis was performed using statistical software SPSS 18.0.0. (SPSS Inc. Chicago, IL, USA). *P*-values (p) less than 0.05 were considered significant. Shapiro-Wilk test was used to examine the normality assumption of continuous variables. Descriptive statistics were reported as mean ± SD for quantitative variables and were summarized by number and percentages for qualitative variables. Quantitative variables were compared with students’ t-test between the two groups. Spearman and Pearson correlation tests were applied for examining the association between two quantitative variables.

Multiple linear regression analysis was used to determine the parameters most predictive of the salivary CSTB and DMBT1. A stepwise forward regression algorithm was applied to select parameters to be entered in the final model. All variables which were significant in univariate analysis and biologically plausible to affect the continuous outcomes (salivary CSTB and DMBT1) were selected to be evaluated in the aforementioned algorithm. Only the variables that entered the model at *p*-values less than 0.1 were included in the final model. Univariate and multivariable logistic regression analyses were conducted to examine the association between the explanatory variables and the presence of GC.

Receiver operating characteristic (ROC) curve was constructed to assess the diagnostic values of salivary CSTB and DMBT1 for differentiating GC patients from healthy controls. MedCalc® Statistical Software version 19.8 (MedCalc Software Ltd., Ostend, Belgium; https://www.medcalc.org; 2021) was used to construct ROC curve and to find optimal cut-off value.

### Machine learning method

To assess the effectiveness of CSTB and DMBT1 for GC prediction, we perform a set of machine learning analyses. For this aim, we extract demographic data, clinical characteristics, and food intake status features in addition to CSTB and DMBT1. Also, we use another feature during the experiments, namely α which is derived as:

$$\upalpha =\frac{\mathrm{DMBT}1}{\mathrm{CSTB}}$$

We used an artificial neural network as a supervised machine learning method to predict GC. A multi-layer fully connected feed-forward neural network method was constructed to predict the label of data samples. For implementing the proposed model, we used Python Software Foundation, Version 3.7, and the Keras library [58], which is a high-level neural network API. During the training phase of the constructed method, for each training data sample, the extracted features, including DMBT1, CSTB, alpha (α) besides demographics, clinical characteristics and food intake status features, were normalized in the range of [0, 1] and were entered into the network. The output value indicates the label of the data sample. 80% of data samples were used during the training and validation phase, and 20% remaining were used during the test phase. We used the 4-fold cross-validation method and the Adam optimizer [59] as the optimization algorithm. Data samples were labeled as follows; GC patients were labeled 1 and control cases were labeled 0. The constructed model has two hidden layers, and each hidden layer has 16 neurons. We used the ReLU activation function for the hidden layers, and the sigmoid activation function was used for the output layer. We also used binary cross-entropy as the loss function of the artificial neural network.

## Results

### Patient characteristics

Demographics, clinical characteristics, and laboratory findings of patients with GC and healthy controls are summarized in Table 1. The two groups differed significantly according to educational level (*p* < 0.0001), occupational status (*p* < 0.0001), positive history of GERD (*p* < 0.0001), positive history of gastric ulcers (*p* = 0.01), vegetable consumption (*p* = 0.02) and salty taste preference (*p* = 0.02) (Table 1).

**Table 1:** Demographics, clinical characteristics, food intake status, and laboratory findings of patients with GC and healthy controls

Characteristics	GC patients (***n*** = 31)	Healthy controls (***n*** = 31)	p^*^
*Gender*			0.12
Female	4 (12.90%)	9 (29.03%)	
Male	27 (87.10%)	22 (70.97%)	
*Age, years*	63.42 ± 11.40	59.06 ± 7.78	0.08
*Education levels*			< 0.0001
Primary and secondary	28 (90.32%)	13 (41.94%)	
Diploma and BSc.	3 (9.68%)	13 (41.94%)	
MSc. and PhD.	0 (0.00%)	5 (16.13%)	
*Occupational stress levels*			< 0.0001
Low stress level	6 (19.35%)	26 (83.87%)	
Moderate stress level	23 (74.19%)	3 (9.68%)	
High stress level	2 (6.45%)	2 (6.45%)	
*Positive history of disease*			
GERD	13 (41.94%)	21 (67.74%)	0.04
Gastric ulcers	7 (22.58%)	0 (0.00%)	0.01
Anemia	2 (6.45%)	0 (0.00%)	0.49
*Types of patient care*	–	–	–
Inpatient	7 (22.58%)	–	–
Outpatient	24 (77.42%)	–	–
*Current smoking status*	9 (29.03%)	10 (32.26%)	0.78
*Drug consumption*	3 (9.68%)	4 (12.90%)	1.00
*Alcohol consumption*	1 (3.23%)	1 (3.23%)	1.00
*History of radiotherapy*	0 (0.00%)	0 (0.00%)	–
*Vegetable consumption*	16 (51.61%)	25 (80.65%)	0.02
*Fast food consumption*	0 (0.00%)	1 (3.23%)	1.00
*Salty fish consumption*	2 (6.45%)	0 (0.00%)	0.49
*Salty taste preference*	12 (38.71%)	4 (12.90%)	0.02
*Sour taste preference*	8 (25.81%)	6 (19.35%)	0.54
*Spicy taste preference*	9 (29.03%)	8 (25.81%)	0.78
*Salivary CSTB level, ng/mL*	115.55 ± 7.06	128.30 ± 18.06	0.001
*Salivary DMBT1 level, ng/mL*	171.88 ± 39.67	139.76 ± 39.05	0.002

Values are expressed as mean ± SD or No. (%).

* *P*-value (p) for the comparison between patients with GC and healthy control groups

However, no statistically significant difference was observed between patients with GC and healthy controls regarding gender, age, positive history of anemia, current smoking status, drug consumption, alcohol consumption, fast food consumption, salty fish consumption, and sour and spicy taste preferences (Table 1).

### Salivary CSTB and DMBT1 concentrations

The mean salivary CSTB level was significantly lower in GC patients in comparison with healthy controls (*p* = 0.001, Table 1). The mean DMBT1 concentration was significantly higher in GC patients compared with healthy controls (*p* = 0.002, Table 1).

### Association between salivary CSTB levels and all evaluated variables

Table 2 summarizes the mean salivary CSTB concentrations according to demographics, clinical characteristics, and food intake status of participants in each study group. Spearman correlation test resulted in a significant positive association between age and salivary CSTB levels in healthy controls (*r* = 0.36 and *p* = 0.046). However, no significant correlation was found between these two parameters in the patient group (*r* = 0.32 and *p* = 0.08). In both study groups, no association was observed between salivary CSTB levels and other evaluated parameters in Table 2. According to the results of multiple linear regression, GC was significantly associated with low levels of salivary CSTB after controlling the effects of age of participants (adjusted R^2^ = 0.21, F = 8.96, and *p* < 0.001).

**Table 2:** Salivary CSTB and DMBT1 levels according to demographics, clinical characteristics, and food intake status of study groups

	Salivary CSTB level (ng/mL)		DMBT1 level (ng/mL)	
Characteristics	Patients with GC (*n* = 31)	Healthy Controls (*n* = 31)	Patients with GC (*n* = 31)	Healthy Controls (*n* = 31)
Gender				
*Female*	113.52 ± 4.27	135.21 ± 20.07	166.50 ± 35.23	134.22 ± 28.52
*Male*	115.86 ± 7.40	125.47 ± 16.84	172.68 ± 40.83	142.03 ± 43.01
*p-value*^*#*^	0.40	0.22	0.76	0.56
Education				
*Primary and secondary*	115.67 ± 7.41	134.35 ± 14.06	169.84 ± 40.05	149.13 ± 40.76
*Diploma and above*	114.48 ± 2.37	123.92 ± 19.69	190.89 ± 36.69	133.00 ± 37.46
*p-value*^*#*^	0.56	0.10	0.43	0.27
Occupation				
*Low stress*	114.74 ± 3.81	129.96 ± 17.32	179.72 ± 34.17	136.86 ± 39.31
*Moderate-to-high stress*	115.75 ± 7.69	119.62 ± 21.44	170.00 ± 41.29	154.87 ± 37.95
*p-value*^*#*^	0.56	0.36	0.56	0.37
History of GERD				
*Yes*	115.24 ± 4.22	130.12 ± 18.47	173.87 ± 31.89	145.95 ± 43.24
*No*	115.78 ± 8.68	124.47 ± 17.47	170.44 ± 45.32	126.77 ± 25.52
*p-value*^*#*^	0.56	0.42	0.81	0.13
History of gastric ulcers				
*Yes*	116.21 ± 7.80	–	168.86 ± 39.08	–
*No*	115.21 ± 7.80	128.30 ± 18.06	172.76 ± 40.63	139.76 ± 39.05
*p-value*^*#*^	0.48	–	0.82	–
Current smoking status				
*Yes*	115.66 ± 4.00	126.22 ± 17.78	177.56 ± 59.71	140.97 ± 33.38
*No*	115.51 ± 8.08	129.29 ± 18.54	169.56 ± 29.50	139.19 ± 42.25
*p-value*^*#*^	0.95	0.66	0.71	0.90
Drug consumption				
*Yes*	109.58 ± 19.24	124.38 ± 25.29	140.78 ± 58.58	160.33 ± 32.04
*No*	116.19 ± 4.86	128.88 ± 17.31	175.21 ± 37.08	136.72 ± 39.58
*p-value*^*#*^	0.61	0.75	0.42	0.25
Vegetable consumption				
*Yes*	114.57 ± 8.40	130.10 ± 18.21	169.42 ± 47.02	139.31 ± 41.73
*No*	116.60 ± 5.40	120.78 ± 16.74	174.51 ± 31.45	141.67 ± 28.05
*p-value*^*#*^	0.43	0.26	0.72	0.87
Salty taste preference				
*Yes*	116.88 ± 2.97	122.58 ± 28.86	163.97 ± 46.90	143.75 ± 21.21
*No*	114.72 ± 8.71	129.14 ± 16.56	176.88 ± 34.79	139.17 ± 41.29
*p-value*^*#*^	0.33	0.68	0.42	0.74
Sour taste preference				
*Yes*	114.92 ± 12.19	128.12 ± 20.83	163.67 ± 30.18	119.00 ± 40.65
*No*	115.77 ± 4.54	128.34 ± 17.81	174.74 ± 42.70	144.75 ± 37.79
*p-value*^*#*^	0.85	0.98	0.44	0.20
Spicy taste preference				
*Yes*	117.95 ± 5.61	120.59 ± 17.94	173.41 ± 45.27	126.88 ± 25.00
*No*	114.57 ± 7.47	130.98 ± 17.70	171.26 ± 38.28	144.25 ± 42.42
*p-value*^*#*^	0.18	0.18	0.90	0.18

Data are expressed as mean ± SD

# *p*-value (p) to compare the mean outcomes (salivary CSTB and DMBT1) between the categories of each characteristic in each study group

### Association between DMBT1 level and all other evaluated variables

The mean DMBT1 levels according to demographics, clinical characteristics, and food intake status of participants in each study group are presented in Table 2. No significant correlation was observed between the age of participants and DMBT1 levels in both groups (*r* = 0.33 and *p* = 0.07 in patients; *r* = 0.28 and *p* = 0.13 in controls). In both groups, no association was found between DMBT1 concentration and each of the other explanatory variables in Table 2. Multiple linear regression analysis demonstrated that GC was significantly correlated with high levels of DMBT1 after controlling the effects of the age of participants (adjusted R^2^ = 0.20, F = 8.67, and *p* < 0.001).

### Association between salivary CSTB and DMBT1 levels

There was no significant association between salivary CSTB and DMBT1 levels in patients with GC (*r* = − 0.04 and *p* = 0.85) and healthy controls (*r* = − 0.18 and *p* = 0.33).

### Association between evaluated variables and the risk of developing gastric cancer

Based on univariate binary logistic regression, participants with higher educational level (odds ratio (OR) = 0.08; 95% confidence interval (CI) = 0.02 to 0.31), with lower stress jobs (OR = 0.04; 95% CI = 0.01 to 0.17) and with higher consumption of vegetables (OR = 0.26; 95% CI = 0.08 to 0.80) were less likely to develop GC. Salty taste preference was significantly associated with risk of GC (OR = 4.26; 95% CI = 1.19 to 15.25). Individuals with a positive history of GERD were less likely to develop GC than those without a positive history of GERD (OR = 0.34; 95% CI = 0.12 to 0.97). However, developing GC did not significantly correlate with age, gender, current smoking status, and sour and spicy taste preferences (Table 3). Individuals had 7% reduction in the risk of GC per one-unit increase in salivary CSTB level (OR = 0.93; 95% CI = 0.88 to 0.97, Table 3). A 0.02-fold increased risk of GC was found per one-unit increase in the level of DMBT1 (OR = 1.02; 95% CI = 1.01 to 1.04, Table 3).

**Table 3:** Results of univariate binary logistic regression analysis of the association between evaluated variables and the risk of developing GC

Characteristics	Crude OR (95% CI for OR)	p
Age	1.05 (0.99 to 1.10)	0.09
Gender (Female vs. male)	0.36 (0.10 to 1.34)	0.13
Education (Diploma and higher vs. primary and secondary)	0.08 (0.02 to 0.31)	< 0.001
Occupation (Low vs. moderate-to-high stress level)	0.04 (0.01 to 0.17)	< 0.0001
Positive history of GERD	0.34 (0.12 to 0.97)	0.04
Positive history of gastric ulcers^a^	–	–
Positive history of anemia^a^	–	–
Current smoking status	0.86 (0.29 to 2.53)	0.78
Drug consumption	–	–
Alcohol consumption^a^	–	–
Vegetable consumption	0.26 (0.08 to 0.80)	0.02
Fast food consumption^a^	–	–
Salty fish consumption^a^	-	–
Salty taste preference	4.26 (1.19 to 15.25)	0.03
Sour taste preference	1.45 (0.44 to 4.81)	0.54
Spicy taste preference	1.18 (0.38 to 3.60)	0.78
Salivary CSTB, ng/mL	0.93 (0.88 to 0.97)	0.003
Salivary DMBT1, ng/mL	1.02 (1.01 to 1.04)	0.005

^a^ There was insufficient data for statistical analysis

According to multivariable logistic regression analysis, salivary CSTB level (OR = 0.89; 95% CI = 0.81 to 0.98, *p* = 0.1), DMBT1 level (OR = 1.02; 95% CI = 1.00 to 1.05, *p* = 0.05), occupation status (OR (low vs. moderate-to-high stress level) = 0.07; 95% CI = 0.01 to 0.40, *p* = 0.003) and educational level (OR (Diploma and higher vs. primary and secondary) = 0.03; 95% CI = 0.003 to 0.29, *p* = 0.003) were the significant determinants of developing GC.

### The ROC curve for differentiating gastric cancer patients from healthy controls

**Fig. 2:**
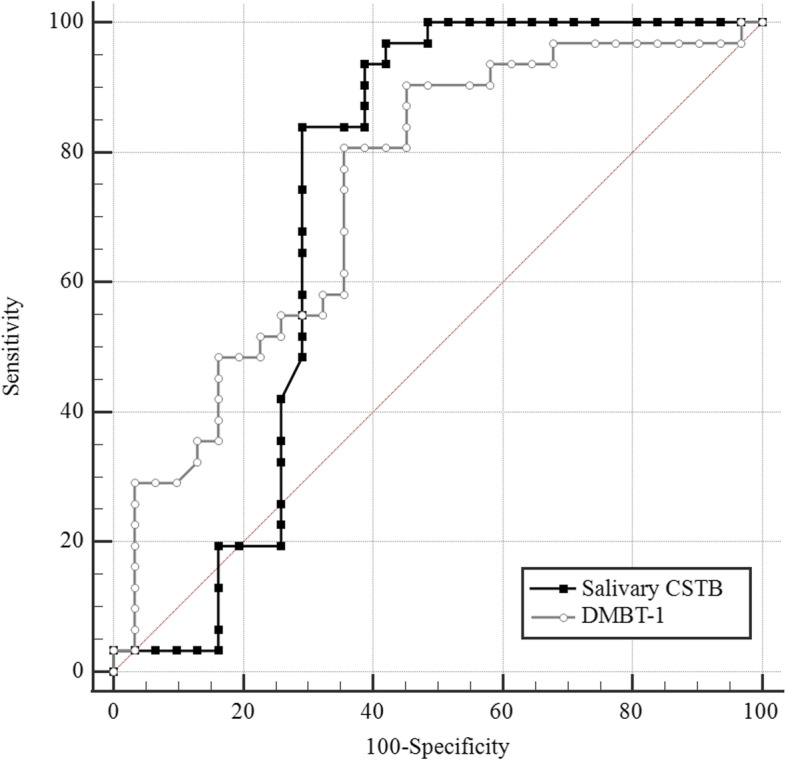
ROC curve for DMBT1 (AUC = 0.741, sensitivity = 80.65%, specificity = 64.52%) and ROC curve for salivary CSTB (AUC = 0.728, sensitivity = 83.87%, specificity = 70.79%)

ROC curve was constructed to estimate diagnostic values of DMBT1 for differentiating GC patients from healthy controls. The results showed that the area under the ROC curve was up to 0.741 (95% CI = 0.614 to 0.844; *p* < 0.001and Fig. 2). The optimal cut-off value for differentiating GC patients from healthy controls was DMBT1 levels greater than 146.33 ng/mL with which the sensitivity and specificity were 80.65 and 64.52%, respectively.

**Fig. 3:**
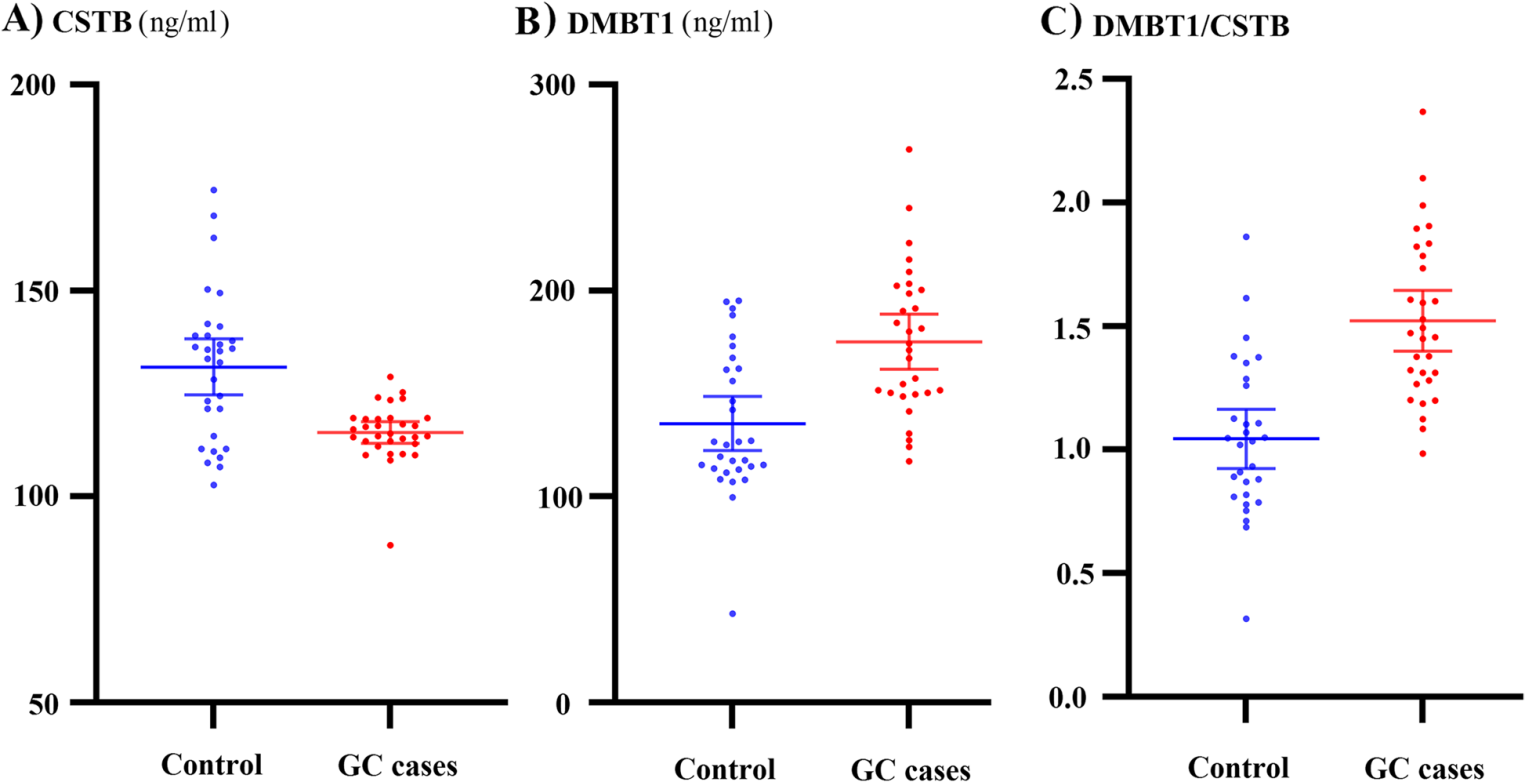
**A** Salivary CSTB levels in GC patients and control (*p* < 0.001), **B** salivary DMBT1 levels in GC patients and control (*p* < 0.001), and **C** salivary DMBT1/CSTB levels in GC patients and control (*p* < 0.001)

In addition, a ROC curve was constructed to estimate diagnostic values of salivary CSTB. The optimal cut-off point for differentiating GC patients from healthy controls was salivary CSTB levels equal to or lower than 119.06 ng/mL. At this cut-off point, the sensitivity and specificity were 83.87 and 70.97%, respectively. The area under the ROC curve was calculated as 0.728 (95% CI = 0.60 to 0.83; *p* = 0.002). Comparing the accuracy of salivary CSTB and DMBT1 in detecting GC indicated no significant difference between these diagnostic tests for differentiating the GC patients from healthy individuals (difference between areas = 0.01, 95% CI = − 0.170 to 0.19; *p* = 0.89, Fig. 2). The optimal cut-off point of α (DMBT1/CSTB) for distinction GC patients from controls was equal to or lower than 1.157 (Fig. 3). At this cut-off point, the sensitivity and specificity were 90.00 and 72.41%, respectively.

### Machine learning analysis

In order to analyze the DMBT1, CSTB and α features, we constructed five models with various input feature vectors, in which the values of each feature vector were normalized using the robust scaler method as follows:

$$Normalized\ value=\frac{value- median\ of\ feature\ vector\ (i)}{IQR\ of\ feature\ vector\ (i)},1\le i\le 19$$

**Fig. 4:**
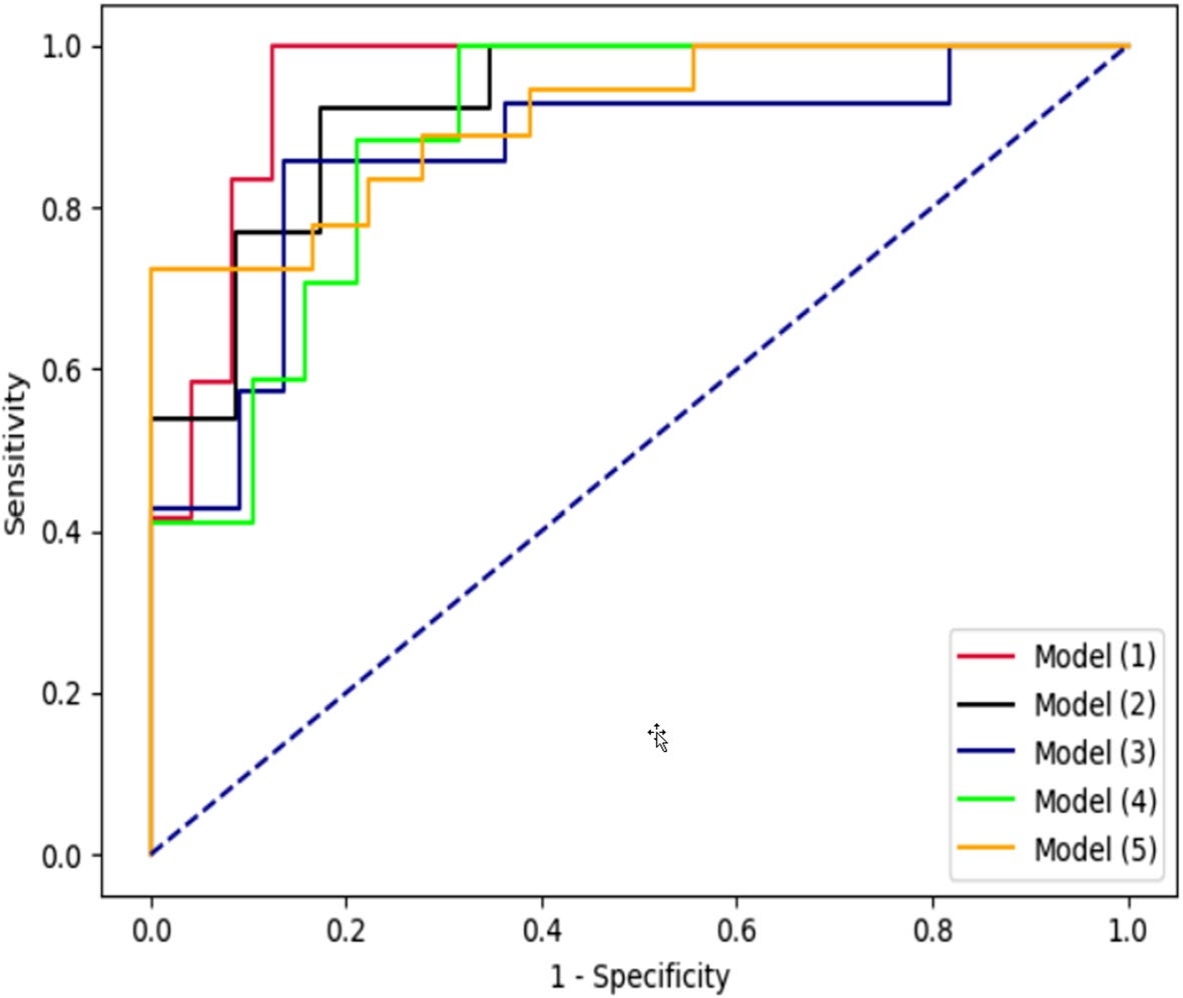
ROC curve analysis of five machine learning models

IQR is the range between the first quartile and the third quartile. The input feature vector of the model (1) includes all the extracted features including gender, age (years), education levels, occupational stress levels, positive history of disease, types of patient care, current smoking status, drug consumption, alcohol consumption, history of radiotherapy, vegetable consumption, fast food consumption, salty fish consumption, salty taste preference, sour taste preference, spicy taste preference, salivary CSTB level, salivary DMBT1 level (listed in Table 1), and α; The input feature vector of the model (2) includes all the extracted features, except DMBT1 and α; The input feature vector of the model (3) includes all the extracted features, excluding CSTB and α; The input feature vector of the model (4) includes all the extracted features, excluding DMBT1, CSTB, and α; The input feature vector of the model (5) merely includes DMBT1, CSTB, and α. ROC curve analysis of all models is depicted in Fig. 4.

**Fig. 5:**
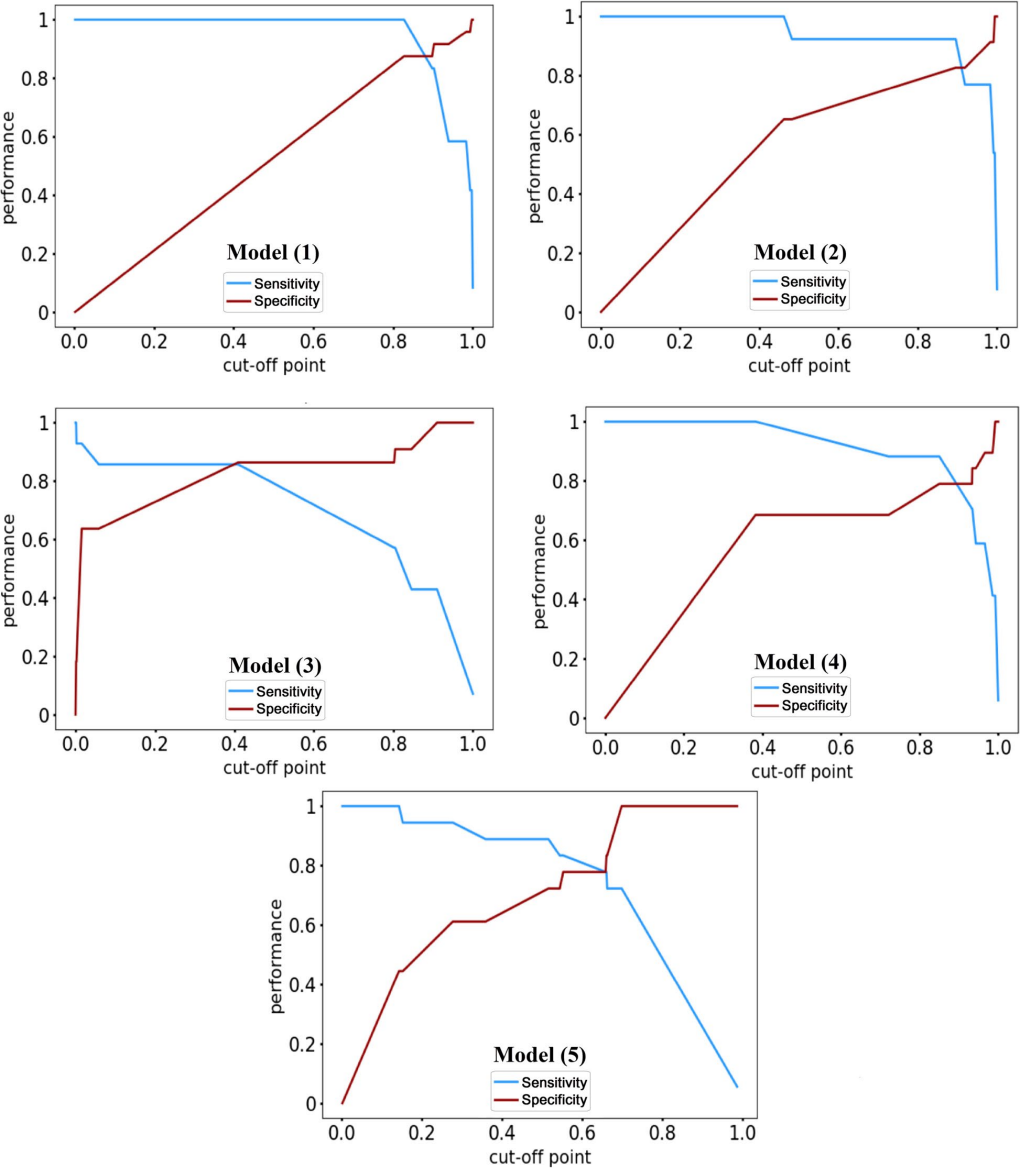
ROC curve analysis of five machine learning models

The area under ROC curve (AUC) of the models revealed that model (1) achieves the highest AUC with the value of 0.95. Elimination of DMBT1, CSTB, and α in the input feature vector causes the AUCs of models (2) and (3) to be reduced by 0.02 and 0.09, respectively. The AUC of the model (4), wherein the DMBT1, CSTB, and *α* features have been removed from the input feature vector, is 0.89. Our analysis shows that the AUC of the model (5), in which the input feature vector is composed of DMBT1, CSTB, and features compared to the first model, merely reduces by 0.04, which indicates the effectiveness of these three features in GC diagnosing. Figure 5 compares the sensitivity and specificity of constructed models on different cut-off point values.

Table 4 summarizes the sensitivity, specificity, positive predictive value (PPV), negative predictive value (NPV), accuracy, and AUC measures of constructed models. For this experiment, we set the cut-off point value for all the models to 0.3 to increase the sensitivity and reduce the prediction error of patient cases.

**Table 4:** Comparison of sensitivity, specificity, PPV, NPV, accuracy, and AUC machine learning models

Measure	Model (1)	Model (2)	Model (3)	Mode (4)	Model (5)
Sensitivity	100%	100%	85.7%	100%	88.8%
Specificity	70.8%	65.2%	86.3%	68.4%	61.1%
PPV	63.0%	6.01%	80.0%	73.9%	69.5%
NPV	100%	100%	90.4%	100%	84.6%
Accuracy	80.5%	77.0%	86.1%	83.3%	75.0%
AUC	0.95	0.93	0.86	0.89	0.91

## Discussion

GC is one of the major health problems in the world. Most cases of GCs are diagnosed in the later stages of the disease and become symptomatic in an advanced stage, while there is no formal screening program for the diseases. Although several screening approaches have been proposed, such as detecting gastric mucosal atrophy by measuring pepsinogens in the bloodstream, none of these methods are usually applied due to the nature of the disease and the deterioration of patients [60, 61]. Despite potential preventive measures and screening methods such as PET-CT and endoscopy, no effective method has been proposed for future clinical trials to reduce GC [62, 63]. Endoscopy and biopsy of the gastric remain the standard diagnostic criteria for GC [62, 64]. Due to the invasiveness of this method and the high cost and difficult access to this diagnostic method, endoscopy cannot be considered a suitable method for screening GC [65].

Biomarkers may serve as a non-invasive diagnosis in the early detection of GC, but due to the nature of GC, no specific and sensitive biomarkers are yet available [66]. It is possible to determine these biomarkers in blood, urine, and saliva that saliva can provide the appropriate way to detect patients, better prognosis, prevent recurrence, and control patient mortality [67]. Several studies have used salivary proteins as potential diagnostic markers to monitor disease, prognosis, patient survival, and treatment [68–70]. It has also been shown that there are blood transfusions in saliva; therefore, it is almost equal to serum [71, 72].

CSTB is a protease inhibitor of cathepsin that is increased in cancer and acts as an intracellular thiol protease inhibitor [73, 74]. Evidence suggests the role of CSTB in various diseases [46, 75]. Animal models have been shown to increase the expression of CSTB inhibiting GC metastasis by involving biological processes involved in proliferation, apoptosis, and migration [76]. Overexpression of CSTB suppresses activation of the PI3K/Akt/mTOR pathway. PI3K/Akt/mTOR pathway is widely involved in regulating cell processes, including angiogenesis, cell proliferation and metabolism [46, 77]. CSTB Downregulation promotes the development and progression of GC by affecting cell proliferation and migration. Previous studies have shown that CSTB plays different roles in ovarian cancer [78, 79], colon cancer [44], and myoclonic epilepsy [80].

This study indicated that salivary CSTB in GC patients was significantly lower than in the control group. Furthermore, this biomarker had an acceptable sensitivity (83.87%) and specificity (70.97%) in GC differentiation from the healthy control. Previous studies have shown that CSTB downregulates both protein and mRNA levels in GC and can be used as a marker in GC diagnosis [25]. Xiao et al. examined the salivary proteome of patients with GC. Five proteins were selected for further study, including interleukin-1 receptor antagonist (ILIRA), CSTB, isomerase triphosphate (TPI1) and DMBT1. ELISA examination of these proteins showed that their expression varied significantly in GC patients and healthy individuals with 85% sensitivity and 80% specificity in diagnosing GC [81].

The DMBT1 gene encodes a protein involved in cell proliferation and is considered a tumor suppressor for the brain and epithelial cancer [82–85]. Some studies have shown conflicting results in reducing or increasing the expression of DMBT1 in various cancers [86, 87]. Preliminary studies have shown that DMBT1 is eliminated or reduced in a variety of tumors [88]. DMBT1 mucosal levels increase significantly (2.5-fold) in patients with gastric mucosal dysplasia and atrophic gastric mucosa [48]. An increase was seen in advanced gastritis associated with *Helicobacter pylori* infection. In addition, the increased expression of DMBT1 was observed in precancerous lesions of the gastric mucosa and the role of DMBT1 in gastric carcinogenesis was complex [48, 89]. Conde et al. showed that DMBT1 downregulates mRNA levels in 38% of GC patients and upregulates in 62% of GC patients. Loss of DMBT1 is likely to occur in differentiated GCs, while DMBT1 upregulation occurs in all types of GC [90]. Increased expression of DMBT1 in GC was shown in several studies, which confirms our results. Considering the acceptable sensitivity and specificity of salivary DMBT1 in GC detection, DMBT1 may be suggested as a noninvasive marker in GC detection.

Our results showed a significant relationship between consumption of a diet containing fruits and vegetables with GC. Thus, low consumption of vegetables and fruits is associated with an increased risk of GC. These results are in line with Wang et al., who stated that high fruit intake might decrease the risk of non-cardia GC [91]. According to ours, there is a relationship between salty taste preference and GC. Lin et al., in their study, stated that salt taste preference in the diet showed a dose-response relationship with GC. Reducing salt and salt processed food in diets might be one practical measure to preventing GC [57]. Yang et al. stated a significant relationship between salt taste sensitivity threshold and GC [92]. Excessive consumption might act as a gastric mucosa stimulant, leading to atrophic gastritis, increased DNA synthesis, and cell proliferation, thereby providing the basis for GC incidence [4]. Our study indicated that higher consumption of vegetables was less likely to develop GC; this result is confirmed in several studies [93–96].

According to our results, a higher educational level is associated with a lower incidence of GC. Lower educational level is accompanied by risk factors such as *Helicobacter pylori* infection and lifestyle factors such as dietary habits, obesity, and cigarette smoking, which may increase the risk of GC [97–99]. These results are in line with Rota et al. and Lagergren et al. showed that the high level of education was associated with a modest decrease in the GC rate [100, 101].

Individuals with a positive history of GERD were less likely to develop GC than those without a positive history of GERD. These results are in contrast to other studies. They stated that a history of GERD is a risk factor for cardiac GC, which arises from dysplastic intestinal metaplasia, and one potentially involving dysplasia of the cardiac-type mucosa [22, 102–104]. One reason for the difference is the type of cancer examined in the present study and the low sample size compared with other studies.

Participants were classified regarding occupation in three groups; low-stress level, moderate-stress level, high-stress level. Participants with lower stress jobs were less likely to develop GC. These results were in line by Kuwahara et al. results [55]. Also, Eguchi et al. stated that individuals working in coal and tin mining, metal processing (particularly steel and iron), and rubber manufacturing industries had increased risks of GC [105]. Yoshinaga revealed that occupations and industries still impact men’s and women’s health in terms of mortality due to GC in Japan [106].

The sensitivity of CSTB in GC diagnosis is 83.87%, and its specificity is 70.97%. AUC is close to one, and it can be concluded that this protein has an acceptable function in diagnosing GC. Yang et al. examined serum markers for the diagnosis of GC. They showed COPS2, CTSF, NT5E, and TERF1 biomarkers with 95% diagnostic sensitivity and 92% specificity for differentiating GC patients from healthy individuals. They concluded that these four serum biomarkers could be used as a non-invasive diagnostic indicator for GC, and a combination of them could potentially be used as a predictor of overall GC survival [107].

In this study, in addition to studying demographic information and salivary level of CSTB and DMBT1, the relationship between demographic data by taking the Salivary CSTB and DMBT1 into account was investigated to diagnose GC. Applying the information mentioned above to a set of machine learning methods confirmed our achieved findings. Utilizing machine learning methods in cancer diagnosis improves diagnostic accuracy and introduces novel and complex cause-and-effect relationships, which is not easily possible by examining and receiving a patient’s history [108–110]. Hirasawa et al. used a neural network for detecting GC in endoscopic images. They correctly diagnosed GC lesions with a sensitivity of 92.2% and a positive predictive value of 30.6% [54]. Although several studies have used machine learning and artificial intelligence to interpret patients’ images to diagnose GC, the use of machine learning to analyze biomarkers as well as patient demographics has been limited.

Machine learning methods do not cause crucial factors to diagnose GC but help us develop computer algorithms that can consider a set of variables and their complicated relationship. Machine learning is known as the most common engine of artificial intelligence. By taking advantage of machine learning in clinical issues, many useful facilities in public health are provided. The best model of Liu et al. exactly predicted the risk of early GC with the accuracy of 77.84% and the AUC of 0.66 by data mining method of patients’ demographic data using C5.0 decision tree algorithm [111]. Zhu et al. used machine learning analysis of demographic data in the diagnosis of GC. They stated that machine learning is a non-invasive method with a sensitivity of 87.0, specificity of 84.1, and AUC equal to 0.91 for GC diagnosis, reducing medical costs [112]. These results are in accord with ours, indicating the ability of machine learning to analyze demographic data.

Aslam et al. showed that using machine learning and support vector machine (SVM) for analyzing the results of high-performance liquid chromatography-mass spectrometry (HPLC-MS) of saliva led to an overall accuracy of 97.18%, specificity of 97.44%, and sensitivity of 96.88% for the diagnosis of GC [113]. In this study, in addition to statistical analysis of the salivary CSTB and DMBT1, using various machine learning methods, we simultaneously analyzed the CSTB and DMBT1 salivary levels as a non-invasive method as well as demographic data, clinical characteristics, and nutrition habits of patients and control group.

## Conclusion

This study was designed to evaluate the salivary expression levels of CSTB and DMBT1 in GC patients with healthy individuals. Using statistical analysis and various machine learning models based on the salivary CSTB and DMBT1 concentrations, demographic, clinical characteristics data, and nutrition habits, differentiation criteria for detecting GC patients from healthy control were proposed. This study showed a significant difference between salivary expression levels of CSTB and DMBT1 proteins in healthy individuals and GC patients. The expression of CSTB in the saliva of patients with GC decreased significantly compared to its expression in the saliva of healthy individuals. The salivary expression levels of DMBT1 increased in GC cases rather than healthy control significantly. These two diagnostic biomarkers expressed in saliva can probably be used as a non-invasive method in GC’s early diagnosis and prognosis. Among the demographic factors, education levels, and occupational stress levels; Among the clinical characteristics data, history of GERD and the history of gastric ulcers; Among the food intake habits, vegetable consumption, and salty taste preference, there is a significant difference between GC case and control. Various machine learning analyses using biomarkers, demographic, clinical and nutrition habits data could provide affordability offer models with acceptable accuracy for differentiation of GC and control by a cost-effective and non-invasive method.

## Data Availability

The datasets used and/or analyzed during the current study are available from the corresponding author on reasonable request. Machine learning code that was used is accessible via the following address: https://codeberg.org/mansur/CSTB_and_DMBT1_gastric_cancer.git.

## Abbreviations

AUC: Area under ROC curve
CPD: Cigarettes per day
CT: Computed tomography
CI: Confidence interval
CSTB: Cystatin B
DMBT1: Deleted in malignant brain tumor 1
ELISA: Enzyme-linked immunosorbent assay
FS: Frequency score
GC: Gastric cancer
GERD: Gastroesophageal reflux disease
HPLC-MS: High-performance liquid chromatography-mass spectrometry
MRI: Magnetic resonance imaging
NPV: Negative predictive value
OR: Odds ratio
PPV: Positive predictive value
PET: Positron emission tomography
ROC: Receiver operating characteristic
SRCR: Scavenger receptor cysteine-rich
SVM: Support vector machine
